# Genome Sequence of *Pseudomonas stutzeri* 273 and Identification of the Exopolysaccharide EPS273 Biosynthesis Locus

**DOI:** 10.3390/md15070218

**Published:** 2017-07-10

**Authors:** Shimei Wu, Rikuan Zheng, Zhenxia Sha, Chaomin Sun

**Affiliations:** 1 College of Life Sciences, Qingdao University, Qingdao 266071, China; shimeiwu2016@126.com; 2 Key Laboratory of Experimental Marine Biology, Institute of Oceanology, Chinese Academy of Sciences, Qingdao 266071, China; zhengrikuan15@mails.ucas.ac.cn; 3 College of Earth Science, University of Chinese Academy of Sciences, Beijing 100049, China; 4 Laboratory for Marine Biology and Biotechnology, Qingdao National Laboratory for Marine Science and Technology, Qingdao 266071, China

**Keywords:** genome, *Pseudomonas stutzeri*, exopolysaccharide, biosynthesis, biofilm

## Abstract

*Pseudomonas stutzeri* 273 is a marine bacterium producing exopolysaccharide 273 (EPS273) with high anti-biofilm activity against *P. aeruginosa* PAO1. Here, the complete genome of *P. stutzeri* 273 was sequenced and the genome contained a circular 5.03 Mb chromosome. With extensive analysis of the genome, a genetic locus containing 18 genes was predicted to be involved in the biosynthesis of EPS273. In order to confirm this prediction, two adjacent genes (*eps273-H* and *eps273-I*) encoding glycosyltransferases and one gene (*eps273-O*) encoding tyrosine protein kinase within the genetic locus were deleted and biosynthesis of EPS273 was checked in parallel. The molecular weight profile of EPS purified from the mutant Δ*eps273-HI* was obviously different from that purified from wild-type *P. stutzeri* 273, while the corresponding EPS was hardly detected from the mutant Δ*eps273-O*, which indicated the involvement of the proposed 18-gene cluster in the biosynthesis of EPS273. Moreover, the mutant Δ*eps273-HI* had the biofilm formed earlier compared with the wild type, and the mutant Δ*eps273-O* almost completely lost the ability of biofilm formation. Therefore, EPS273 might facilitate the biofilm formation for its producing strain *P. stutzeri* 273 while inhibiting the biofilm formation of *P. aeruginosa* PAO1. This study can contribute to better understanding of the biosynthesis of EPS273 and disclose the biological function of EPS273 for its producing strain *P. stutzeri* 273.

## 1. Introduction

Exopolysaccharides (EPSs) secreted by bacteria have a protective function against the harsh conditions of the natural environment, such as predation by protozoa, phage attack, antibiotics, or toxic compounds and osmotic stress etc. Moreover, EPSs also play important roles in cell recognition, adhesion to surfaces, and biofilm formation [1]. Genes for EPS production are often located in chromosomal or plasmid DNA and organized into gene clusters. Sometimes more than one gene cluster that is involved in the biosynthesis of EPSs can be found in the genome of bacteria [2,3]. The gene cluster of EPSs contain several kinds of specific functional genes, such as genes involved in assembling of repeating units, chain length determination, polymerization, exportation, and regulation [4].

Based on biosynthesis and exportation mechanism of EPSs, three pathways have been suggested. For the first pathway, a multifunctional processive glycosyltransferase (GT), the synthase, catalyzes both polymerization and exportation [5]. For the second pathway, ATP-binding cassette (ABC) transporter plays an important role in the exportation of the biosynthesized repeating unit. The third pathway depends on Wzx-Wzy proteins [6]. For the EPSs biosynthesis-dependent on Wzx-Wzy proteins, the repeating unit is catalyzed by successive GTs, and exported outside the cell across the inner membrane by Wzx protein, then subsequently polymerized by Wzy protein through the addition of the growing EPS chain on the outer face of the inner cell membrane [5]. The final translocation across the outer membrane involves a member of the outer membrane polysaccharide export protein family, such as Wza [7].

Bacterial tyrosine kinases are reported to be associated with EPS biosynthesis in bacteria, which is proposed to be a general feature of EPS production [8]. Genes encoding bacterial tyrosine kinases are often associated with genes involved in EPS production [6]. The kinases are known to regulate EPS production by phosphorylating and thereby activating a biosynthetic enzyme in the pathway of EPS production [9].

*Pseudomonas stutzeri* is a Gram-negative, rod-shaped, and non-fluorescent denitrifying bacterium that exhibits metabolic diversity and is widely distributed in both terrestrial and marine environments [10]. Due to their great metabolic characteristics, they have application potentials in fields including environmental pollutant degradation, denitrification, and nitrogen fixation, thus, some of them have attracted great attention [11,12]. In our previous study, we identified a marine bacterium *P. stutzeri* 273, which exhibited strong activity of anti-biofilm and anti-infection against *P. aeruginosa* PAO1 by producing exopolysacchride 273 (EPS273), and its strong anti-biofouling activity in the marine environment was also observed [13], which indicates that EPS273 has a promising potential in combating bacterial biofilm-associated infection and marine biofouling.

A better understanding of the biosynthesis mechanism and regulation of EPS production will facilitate genetic, metabolic, and proteinic engineering to produce more, or tailor-made, EPS [14]. In the present study, in order to investigate the biosynthesis of EPS273 and provide the basis for developing new, efficient methods to produce EPS273 for its future applications, we sequenced the genome of *P. stutzeri* 273, and analyzed the biosynthesis of EPS273 by a computational approach. Furthermore, corresponding gene deletion mutants were also generated to provide functional evidence of the proposed 18-gene cluster involved in EPS273 biosynthesis, and the biological role of EPS273 for its producing stain *P. stutzeri* 273 was also investigated.

## 2. Results

### 2.1. Genome Properties of P. stutzeri 273

The complete genome of *Pseudomonas stutzeri* 273 is composed of one circular chromosome of 5,030,940 bp with a GC content of 60.78%, which is similar to those of previously-reported *P. stutzeri* strains (GC content of 60.3–64.0%). No nitrogen fixation genes (*nif*) or extrachromosomal elements are found. The genome contains 4717 genes with the total length of 4,421,631 bp, which occupies 87.89% of the genome. There are 58 tRNA genes and 12 rRNA in the genome (Table S1), and a total of 3151 genes are classified into 22 clusters of orthologous groups (COG) categories (Table S2).

### 2.2. Insights from the Genome Sequence of P. stutzeri 273

The metabolic networks were analyzed according to Kyoto Encyclopedia of Genes and Genomes (KEGG) analysis. A total of 299 genes, 44 genes, and 252 genes are involved in the pathway of membrane transport, glycan biosynthesis and metabolism, and carbohydrate metabolisms (Table S3). There are many genes encoding proteins responsible for sugar transport, such as sugar transporter, sugar ABC transporter ATPase, and sugar ABC transporter permease, which might be involved in metabolism, biosynthesis, and transport of EPSs. Genes for complete glycolysis, pentose phosphate pathways, oxidative phosphorylation, and citrate cycle were identified by genome analysis, as previously reported for aerobically heterotrophic bacteria. Furthermore, as in other *P. stutzeri* strains, the genome of the strain *P. stutzeri* 273 has complete sets of genes for flagellum synthesis, denitrification, and bacterial chemotaxis. Notably, there are several gene clusters involved in heavy metal resistance in genome of *P. stutzeri* 273, such as resistance to copper, mercury, and nickel, which indicates that *P. stutzeri* 273 has potentials in bioremediation applications in the future.

**Figure 1:**
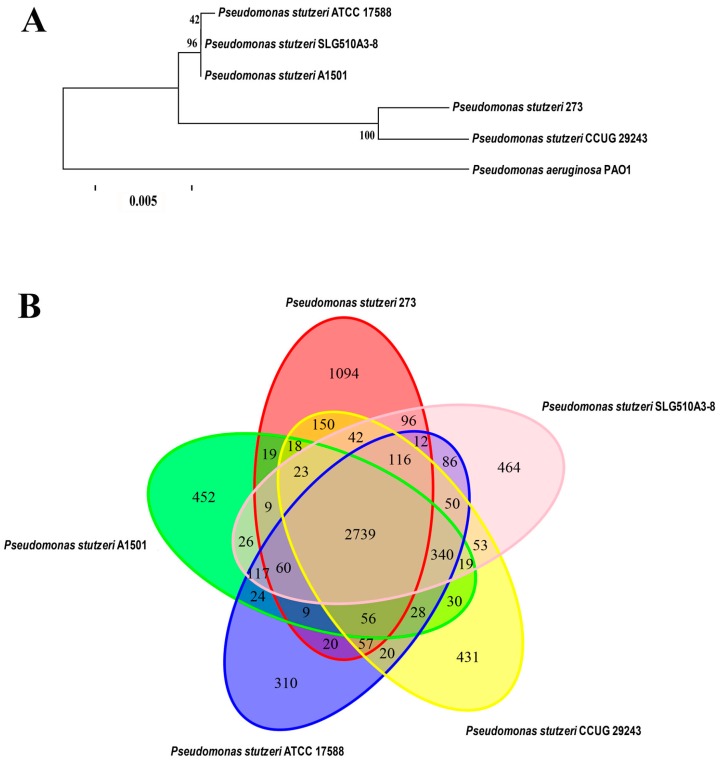
Characterization of *P. stutzeri* 273. (**A**) Phylogenetic tree based on the whole genome of *P. stutzeri* 273, *P. stutzeri* SLG510A3-8, *P. stutzeri* CCUG 29243, *P. stutzeri* ATCC 17588, *P. stutzeri* A1501, and *P. aeruginosa* PAO1. Comparison of above *P. stutzeri* strains was performed using MUMmer [19] and LASTZ [20] alignment tools. The phylogenetic tree was constructed by the TreeBeST [21] using the method of PhyML, and the setting of bootstraps is 1000 with the orthologous genes detected from gene family analysis. The number in the phylogenetic tree represents the reliability of each branch. The bar is 0.005. (**B**) Strain-to-strain variation between *P. stutzeri* 273, *P. stutzeri* SLG510A3-8, *P. stutzeri* CCUG 29243, *P. stutzeri* ATCC 17588, and *P. stutzeri* A1501. The Venn diagram was built using the Vennerable R package. The specific/shared genes of above *P. stutzeri* strains were clustered by the CD-HIT [22] rapid clustering of similar proteins software with a threshold of 50% pairwise identity and 0.7 length difference cutoff in amino acids. The number of genes in different genomes is depicted in the respective ellipse. The number in the overlapping region represents number of genes shared by different genomes. The number in the non-overlapping region represents the number of specific genes owned by different genomes.

Based on the genome sequences, the phylogenetic relationship of *P. stutzeri* 273 with other four *P. stutzeri* strains and *P. aeruginosa* PAO1 was analyzed. As expected, the most distantly phylogenetic neighbor of *P. stutzeri* 273 is *P. aeruginosa* PAO1. In contrast, its closest phylogenetic neighbor is the strain *P. stutzeri* CCUG 29243 (Figure 1A). Notably, *P. stutzeri* CCUG 29243 is a naphthalene-degrading strain isolated from polluted marine sediments of the West Mediterranean Sea [15], and the other three relatively-distant phylogenetic neighbors of *P. stutzeri* SLG510A3-8 [16], *P. stutzeri* ATCC 17588 [17] and *P. stutzeri* A1501 [18] are terrestrial bacteria. Therefore, marine-derived *P. stutzeri* strains might gain some special characteristics during the course of evolution compared with the terrestrial counterparts.

Comparative genomic analyses were also performed among *P. stutzeri* 273, its closely-related phylogenetic neighbor *P. stutzeri* CCUG 29243, and three more distantly-related strains, *P. stutzeri* SLG510A3-8, *P. stutzeri* ATCC 17588, and *P. stutzeri* A1501, which were fully sequenced (Figure 1B). The results of comparative analyses showed that most of the genomic regions were conserved while some differences between the genome of *P. stutzeri* 273 and genomes of other strains also existed. The five strains of *P. stutzeri* share 2739 genes, which make up the core component of the genome in each strain. A total of 1094 genes exist only in *P. stutzeri* 273, which are far more than those that exist strain-specifically in the other four strains of *P. stutzeri*. Notably, a large part of these strain-specific genes of *P. stutzeri* 273 was observed in seventeen putative genomic islands (GIs), which might be involved in “fast forward evolution” of the host. Therefore, analysis of the marine strain *P. stutzeri* 273 will help us gain an insight into the evolution of the species by analyzing members derived from different environments and better understand strain adaptation to marine habitats of *P. stutzeri*.

Notably, except the 2739 genes shared by all five *P. stutzeri* strains, comparative genomic analysis showed that marine derived strains *P. stutzeri* 273 and *P. stutzeri* CCUG 29243 shared an additional 462 genes, which were more than those shared with the other three terrestrial *P. stutzeri* strains. This analysis is consistent with above result of phylogenetic analysis (Figure 1B). In a word, a comparative genome analysis of *P. stutzeri* strains will help us to elucidate the evolutionary adaptation of the members in this species, and also facilitate us to investigate the genomic dynamics of these organisms.

### 2.3. Identification of EPS273 Biosynthesis Locus in the P. stutzeri 273 Genome

Our previous study showed that *P. stutzeri* 273 exhibited strong antibiofilm and anti-infection activities against *P. aeruginosa* PAO1 by producing EPS273 [13]. To find the potential biosynthesis locus of EPS273, the whole genome of *P. stutzeri* 273 was thoroughly analyzed by bioinformatics. A gene cluster containing 18 genes was proposed to be involved in the biosynthesis of EPS273. In order to gain a better understanding of the putative roles of the proteins in this gene cluster, the DELTA-BLAST (Domain-Enhanced Lookup Time-Accelerated BLAST) algorithm of blastp was used [23], which focuses on the domain structure of the proteins and is beneficial to identify the potential biosynthesis locus of EPS273.

**Figure 2:**
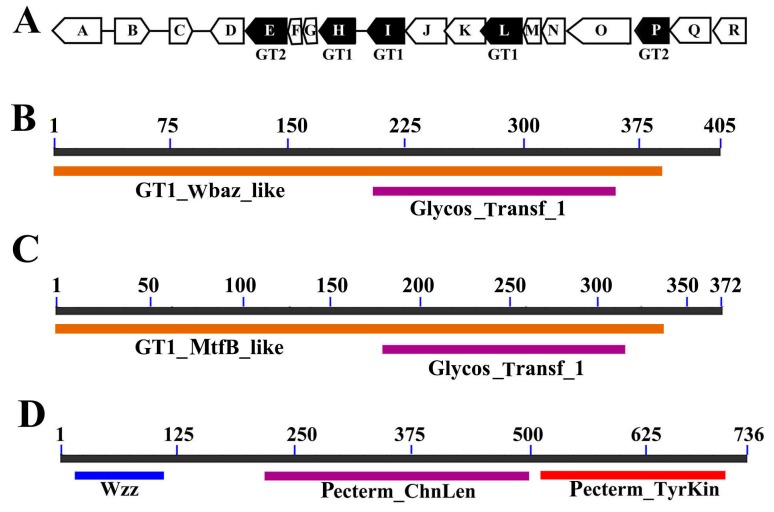
Sequence analyses of the gene cluster encoding EPS273. (**A**) Structure of biosynthesis locus of EPS273. The name for each ORF in the gene cluster of EPS273 is designated as follows: A (EPS273-A), B (EPS273-B), C (EPS273-C), D (EPS273-D), E (EPS273-E), F (EPS273-F), G (EPS273-G), H (EPS273-H), I (EPS273-I), J (EPS273-J), K (EPS273-K), L (EPS273-L), M (EPS273-M), N (EPS273-N), O ( EPS273-O), P (EPS273-P), Q (EPS273-Q), and R (EPS273-R). All the accession numbers are referred to in Table 1; (**B**) The organization of the conserved motifs related to polysaccharide synthesis of the putative family 1 glycosyltransferase EPS273-H; (**C**) The organization of the conserved motifs related to polysaccharide synthesis of the putative family 1 glycosyltransferase EPS273-I. MtfB, mannosyltransferase B; (**D**) The organization of the conserved motifs related to polysaccharide synthesis of the putative protein tyrosine kinase EPS273-O. Pepcterm_ChnLen, polysaccharide chain length determinant protein, PEP-CTERM locus subfamily. The rulers in the Figure 2B–D stand for the amino acids numbers of EPS273-H, EPS273-I and EPS273-O, respectively.

As the first step to analyze the biosynthesis locus of EPS273, genes encoding glycosyltransferases (GTs) were identified. A polysaccharide gene cluster (Figure 2A) consisting of five GTs and 13 other genes were identified, which is similar to the symbiosis exopolysaccharide biosynthetic genes (*syp*) of *Vibrio* sp. reported previously [24]. Within the gene cluster, 14 genes appeared to encode proteins involved in biosynthesis, modification or export of polysaccharides (Figure 2A). According to the analyses, the proteins were grouped into three classes, GTs, polysaccharide exporters, and “other” (Table 1).

Of the 18 putative genes responsible for EPS273 biosynthesis, four genes are predicted to encode regulatory proteins. (i) EPS273-A is a GGDEF domain-containing protein. In molecular biology, the GGDEF domain is a protein domain universally existing in bacteria and is often linked to a regulatory domain, such as a phosphorylation receiver or oxygen sensing domain [25]. The protein containing GGDEF domain exerts its function as a diguanylate cyclase to synthesize cyclic di-GMP, which is an intracellular signaling molecule existing widely in bacteria [25]. Therefore, EPS273-A is proposed to be a diguanylate cyclase for EPS273 biosynthesis. (ii) EPS273-B belongs to P-loop_NTPase superfamily and is predicted to be a sensor kinase. (iii) EPS273-C is a response regulator which contains a signal receiver domain, accepting the signal from the sensor partner of the two-component systems, originally thought to be unique to bacteria (CheY, OmpR, NtrC, and PhoB) [26]. (iv) EPS273-M contains a RfaH domain and is proposed to be the direct transcriptional activator of the EPS273 biosynthesis locus [27].

Of the 14 proposed structural proteins, five are putative GTs, which transfer sugar from an activated donor to a recipient polysaccharide chain. These five proteins, EPS273-H, EPS273-I, EPS273-L, EPS273-E, and EPS273-P, contain conserved domains in the glycosyltransferase 1 (GT1) or glycosyltransferase 2 (GT2) family. EPS273-H, EPS273-I and EPS273-L are identified as family 1 glycosyltransferase. EPS273-H contains a GT1_WbaZ_like domain (Figure 2B), and WbaZ in *Salmonella enterica* has been shown to possess the mannosyl transferase activity [28]. EPS273-I contains a GT1_MtfB_like domain (Figure 2C), and MtfB (mannosyltransferase B) in *E. coli* has been shown to transfer mannose into the growing O9-specific polysaccharide chain [29]. EPS273-L contains a GT1_ExpE7_like domain, and ExpE7 in *Sinorhizobium meliloti* has been shown to be involved in the biosynthesis of galactoglucans [30]. EPS273-E and EPS273-P belong to the glycosyltransferase-like family 2, and members of this family are often involved in bacterial capsule biosynthesis.

One of the EPS273 proteins, a putative polysaccharide exporter EPS273-Q, contains the conserved domain RfbX (COG2244; E value of 2.68 × 10^−15^) found in O-antigen unit translocases, and the RfbX (Wzx) is reported to be involved in transportation of polysaccharide from the cytoplasmic side to the periplasmic side of the cytoplasmic membrane [31].

The remaining eight proteins have putative functions related to polysaccharide modification or chain length regulation. EPS273-D is an acyltransferase, which contains an OafA domain. EPS273-J contains a Glyco_10 domain (E value of 1 × 10^−2^), which is a putative glycosyl hydrolase. EPS273-K is an O-antigen ligase-like membrane protein (E value of 1 × 10^−96^). EPS273-N contains a WbqC domain (E value of 2.14 × 10^−94^). Although this kind of protein containing WbqC domain is functionally uncharacterized, it is reported to be found in an O-antigen gene cluster in *E. coli* and other bacteria, suggesting a role in O-antigen production [32]. EPS273-O is predicted to be a tyrosine protein kinase, which is demonstrated to play an essential role in the biosynthesis of EPS [8]. Moreover, EPS273-O contains a Wzz domain in the N-terminus (E value of 3.98 × 10^−19^) and is predicted to be a chain length determinant protein (Figure 2D). The protein family containing Wzz domain is suggested to play an important role in the distribution of the chain length on the O-antigen component of LPs [9]. EPS273-R is an aminotransferase DegT, which contains a WecE domain (E value of 3.91 × 10^−102^) and is proposed to be a dTDP-4-amino-4,6-dideoxygalactose transaminase [33]. EPS273-F and EPS273-G are two hypothetical proteins and their exact functions need to be elucidated in the future.

Altogether, our analysis suggested that the gene cluster containing the 18 genes identified in *P. stutzeri* 273 might be involved in EPS273 biosynthesis, and corresponding mutants were constructed to confirm this assumption.

### 2.4. Insertional Inactivation of Genes Involved in EPS273 Biosynthesis

**Figure 3:**
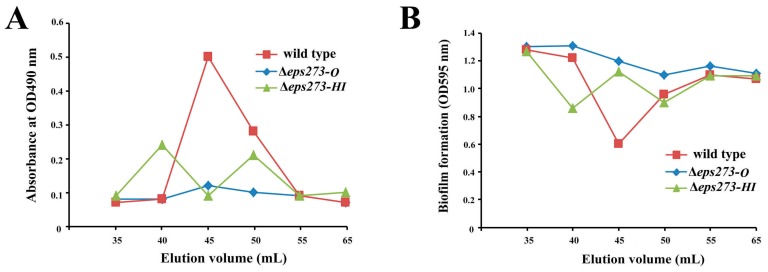
Purification and antibiofilm assays of EPS273 purified from wild type and deletion mutants of *P. stutzeri* 273. (**A**) The profiles of the fractions in the gel filtration, which were collected and monitored for the polysaccharide content determined at OD490 nm after the phenol-sulfuric acid assay. (**B**) The profiles of the fractions in the gel filtration, which were collected and monitored for the biofilm formation determined at OD595 nm after crystal violet staining.

In order to confirm whether the identified 18-gene locus was involved in EPS273 production or not, allelic exchange was used to disrupt genes in this locus as described in the Materials and Methods. Firstly, genes encoding GTs in the proposed 18-gene cluster were selected to be deleted because GTs could transfer sugar from an activated donor to a recipient polysaccharide chain and played an important role in EPSs production. Considering both EPS273-H and EPS273-I belonged to mannosyl transferase according to the DELTA-BLAST analysis and located adjacent in the genome of *P. stutzeri* 273, the two genes encoding EPS273-H and EPS273-I were deleted together from the genome, and the corresponding EPS was purified in parallel with that of wild type strain *P. stutzeri* 273. As for the wild-type strain *P. stutzeri* 273, a polysaccharide peak of EPS273 was eluted immediately after the void volume on Superdex™ 200 column, and showed high antibiofilm activity against *P. aeruginosa* PAO1, which coincided with our previous report [13]. As for the mutant strain Δ*eps273-HI*, two polysaccharide peaks were eluted immediately after the void volume on SuperdexTM 200 column (Figure 3A), which indicated that deletion of *eps273-HI* changed the polysaccharide molecular weight and homogeneity to some extent. Notably, the anti-biofilm activity against *P. aeruginosa* PAO1 was also correspondingly changed (Figure 3B).

Since bacterial tyrosine kinases are reported to be involved in controlling of exopolysaccharide production [8], a gene encoding EPS273-O, a typical tyrosine kinase according to the DELTA-BLAST analysis, was selected to be in-frame deleted by homologous recombination. As shown in Figure 3, the corresponding polysaccharide peak could hardly be detected in mutant strain Δ*eps273-O*, and the anti-biofilm activity against *P. aeruginosa* PAO1 was also reduced dramatically compared with that from the wild-type strain *P. stutzeri* 273 (Figure 3B). Therefore, these results further confirmed that the identified 18-gene locus in *P. stutzeri* 273 was involved in the biosynthesis of EPS273 and EPS273-O played an important role in biosynthesis of EPS273.

### 2.5. The Differences of Colony Phenotype and Biofilm Formation between P. stutzeri 273 and Its Mutants Δeps273-HI and Δeps273-O

**Figure 4:**
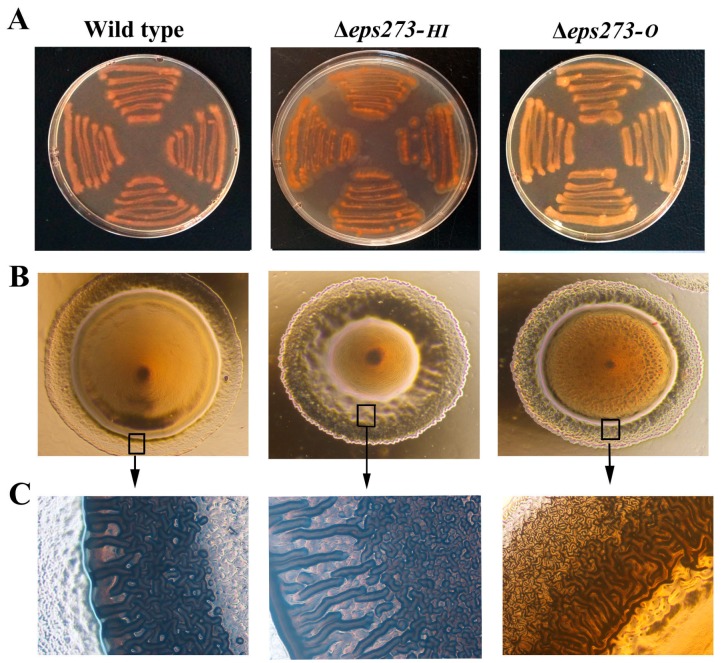
Comparison of colony morphology between wild-type and deletion mutants of *P. stutzeri* 273. (**A**) Congo red binding assay of wild-type *P. stutzeri* 273 compared with deletion mutants Δ*eps273-O* and Δ*eps273-HI*. (**B**) Morphology observation of wild-type *P. stutzeri* 273 compared with deletion mutants Δ*eps273-O* and Δ*eps273-HI*. (**C**) Observation of the edge of the colony in panel B with high magnification. All images are representative of three separate experiments. At least two independent experiments of each strain were tested, and only representative images are shown in panels A–C.

Next, in order to investigate the effect of the gene deletion on the colony phenotype and biofilm formation, the colony phenotype and biofilm formation of the wild-type strain *P. stutzeri* 273 and corresponding deletion mutants Δ*eps273-HI* and Δ*eps273-O* were detected, respectively. As expected, the wild-type strain *P. stutzeri* 273 showed a darker red color on the Congo red plate, while the mutant strain Δ*eps273-O* showed an orange color on the Congo red plate (Figure 4A). This result suggested that the mutant strain Δ*eps273-O* produced less polysaccharide than the wild-type strain did, which was consistent with the results of purification profiles where corresponding EPS273 polysaccharide peak could hardly be detected in mutant Δ*eps273-O* (Figure 3A). However, deletion of *eps273-HI* didn’t change the color of the mutant Δ*eps273-HI* obviously on the Congo red plate, which also coincided with the result of the purification profile that deletion of *eps273-HI* just changed the polysaccharide molecular weight and homogeneity (Figure 4A).

Furthermore, when observed under the microscope, the colonies of mutant strain Δ*eps273-HI* and Δ*eps273-O* looked wrinkled, while colony of the wild type strain *P. stutzeri* 273 was smooth (Figure 4B). Especially, the difference of the colony margins between the wild-type strain *P. stutzeri* 273 and the corresponding mutant Δ*eps273-O* or Δ*eps273-HI* looked more obvious when observed under a microscope (Figure 4C).

**Figure 5:**
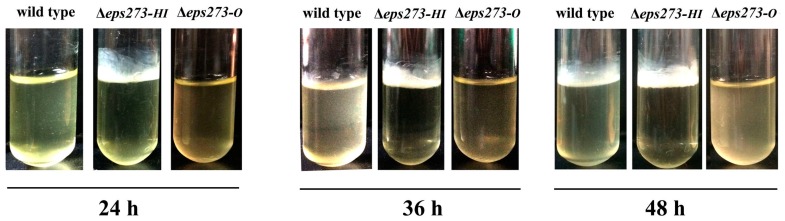
Comparison of the ability of biofilm formation between wild-type *P. stutzeri* 273 and its deletion mutants Δ*eps273-O* and Δ*eps273-HI* at different time points. At least two independent experiments of each strain were performed, and only representative images are shown.

In addition, the mutant strain Δ*eps273-HI* formed the biofilm after incubation for 24 h, while the wild-type strain *P. stutzeri* 273 did not form a biofilm until being incubated for 36–48 h (Figure 5). Surprisingly, the mutant strain Δ*eps273-O* almost completely abolished the ability of biofilm formation as Δ*eps273-O* did not have any biofilm formed even when incubated for up to 48 h (Figure 5). Taken together, these results showed that deletion of *eps273-O* led to a dramatic reduction of biofilm formation due to the reduction of EPS273 production, while deletion of Δ*eps273-HI* facilitated the biofilm formation.

## 3. Discussion

*P. stutzeri* is a species having very broad phenotypic and genotypic diversity. Within prokaryotes, great advances have been achieved in *P. stutzeri*, a species considered as a model system for biochemical characterization of denitrification [17]. In our previous study, *P. stutzeri* 273 was isolated from the sediments of the East China Sea and exhibited great potential in combating biofilm-related infection and biofouling in marine aquaculture [13].

In this study, we sequenced the whole genome of *P. stutzeri* 273. It is noteworthy that 17 GIs were found in the genome of *P. stutzeri* 273. Moreover, genes encoding ABC transporters and genes responsible for resistance to copper and mercury are observed in different GIs. Since GI is predicted to be involved in niche adaptation and the ecological success of microbes by lateral gene transfer [34,35], the 17 GIs in *P. stutzeri* 273 may be important for its adaptation to environmental changes by lateral gene transfer. It will be of great interest to investigate in future studies whether the remaining hypothetical genes present in the GIs of *P. stutzeri* 273 play important roles in conferring its extra resistance to environmental stress, such as heavy metal stress and oxidation stress, etc.

Exopolysaccharide released from bacteria plays important roles in its self-defense of the harsh conditions of starvation, pH, and temperature, and genes responsible for biosynthesis of polysaccharides are often clustered in the genome. Based on bioinformatics analyses, only one complete gene cluster is proposed to be involved in the biosynthesis of EPS273, which is very similar to the *syp* polysaccharide locus reported in *Vibrio fischeri* [23]. Notably, both biosynthesis loci of *syp* and *eps273* include 18 genes that can be grouped into four classes according to putative functions: regulators, glycosyltransferases, export proteins, and proteins with other functions, including polysaccharide modification. The patterns of EPS loci derived from *V. fischeri* and *P. stutzeri* are similar, which indicates that biosynthesis of *syp*-like polysaccharide in different bacteria genus is conserved. Until now, there is no any other report about *syp*-like cluster involved in polysaccharide biosynthesis in *P. stutzeri*. Therefore, it will be interesting to investigate the exact function of each gene in the *eps273* gene cluster and compare it to that of *V. fischeri* counterpart.

As the proposed 18-gene cluster contains five glycosyltransferases, of which EPS273-H and EPS273-I belonged to mannosyl transferase and are located adjacently in the gene cluster, genes encoding EPS273-H and EPS273-I were deleted together. Deletion of *eps273-H* and *eps273-I* together just changed the polysaccharide molecular weight and homogeneity (Figure 3A), indicating that deletion of the two adjacent glycosyltransferases might be complemented by other glycosyltransferases present in the gene cluster, which was consistent with the previous report about expolysaccharide HE800 [24]. To some extent, the presence of five glycosyltransferases within the 18-gene cluster of *P. stutzeri* 273 may confer its ability to generate diversity in the glycosidic structure, which may increase its adaption to the changes of natural environment. In addition, the gene encoding EPS273-O, a tyrosine kinase of *P. stutzeri* 273, was also in-frame deleted. The resulting mutant Δ*eps273-O* almost abolished the production of EPS273 and its biofilm formation (Figure 3 and Figure 5), suggesting that EPS273-O may play an essential role in EPS273 biosynthesis and its biofilm formation, which is in accordance with previous report that bacterial tyrosine kinase is necessary for exopolysaccharide biosynthetic production and biofilm formation in *B. subtilis* [36]. Altogether, the analysis about mutants Δ*eps273-HI* and Δ*eps273-O* further confirmed the involvement of the 18-gene cluster in biosynthesis of EPS273.

To investigate the biological role of EPS273 for its producing strain *P. stutzeri* 273, biofilm of wild-type *P. stutzeri* 273 and its derivative mutants was detected. The deletion mutant Δ*eps273-O* almost completely lost the ability of biofilm formation as the production of EPS273 could hardly be detected, indicating that EPS273 might be important in biofilm formation for its producing strain *P. stutzeri* 273. Together with our previous report that EPS273 exhibited high anti-biofilm activity against *P. aeruginosa* PAO1 [13], it may be presumed that EPS273 might benefit its producing stain *P. stutzeri* 273 to have biofilm formed, and facilitate competition with other strains in niches, because the formation of biofilm is an integral part of life cycle in bacteria and an important factor for bacterial survival in diverse environments [37]. It would be of great interest to elucidate the mechanism of EPS273 to inhibit the biofilm formation of *P. aeruginosa* PAO1 and facilitate its producing strain *P. stutzeri* 273 to form a biofilm. A corresponding study is under way in our lab.

Altogether, the genomic sequence of *P. stutzeri* 273 not only provides us the basic knowledge of the strain, but also helps us to identify EPS273 biosynthesis locus. Combined with results of related gene-deletion mutants, the proposed 18-gene cluster could be claimed to be involved in the biosynthesis of EPS273. This study would help us to design methods to obtain a better production yield, as well as to obtain tailor-made EPS273 with novel properties in the future.

## 4. Materials and Methods

### 4.1. Bacterial Strains and Media

*Escherichia coli* DH5α was used as the host for plasmid construction, and *E. coli* S17-1 was used as a vector donor in conjugation. *P. stutzeri* 273 and corresponding mutants were cultured in marine broth 2216E (5 g/L tryptone, 1 g/L yeast extract, one liter filtered seawater, pH adjusted to 7.4–7.6) or Lysogeny Broth (LB) medium (10 g/L peptone, 5 g/L yeast extract, 10 g/L NaCl, pH adjusted to 7.0), and incubated at 28 °C under vigorous agitation at a speed of 150 rpm. *P. aeruginosa* PAO1, *E. coli* DH5α, *E. coli* SY327, and *E. coli* S17-1 were grown in LB medium at 37 °C with shaking at a speed of 150 rpm. When necessary, antibiotics were added and the final concentrations were used as follows: 25 μg/mL for chloramphenicol (Cm) and 25 μg/mL for gentamicin (Gm).

### 4.2. Genomic Studies

The whole genome of *P. stutzeri* 273 was sequenced by single molecule, real-time (SMRT) technology [38]. Using SMRT Analysis 2.3.0 to filter low-quality reads and the filtered reads were assembled to generate one contig without gaps. A total of 90,551 filtered paired-end reads were produced with an average read length of 12,368 bp, which corresponded to approximately 222-fold coverage. tRNAscan-SE v.1.23 and RNAmmer v.1.2 were used to identify presence of tRNA and rRNA, respectively [39,40]. Gene prediction was performed by GeneMarkS [41] with an integrated model that combined the GeneMarkS generated (native) and heuristic model parameters. A whole genome Blast search (E-value less than 1 × 10^−5^), minimal alignment length percentage larger than 40%) was performed against six databases. They are KEGG (Kyoto Encyclopedia of genes and genomes) [42], COG (Clusters of Orthologous Groups) [43], GO (Gene Ontology) [44], NR (Non-Redundant Protein Database databases), and Swiss-Prot [45]. The Venn diagram was constructed by Vennerable R package [46]. The CAZy family of glycosyltransferases was determined based on the Carbohydrate-Active enZYmes database [47].

### 4.3. Vector Construction and Mutagenesis

The *P. stutzeri* 273 derivatives Δ*eps273-HI* and Δ*eps273-O* were constructed by allelic exchange by the method described previously with minor modification [48]. Briefly, fragments for mutant construction of Δ*eps273-O* were amplified from the strain *P. stutzeri* 273 by primers P1/P2 and P3/P4, and fragments for mutant construction of Δ*eps273-HI* were amplified by primers P5/P6 and P7/P8 (Table S4), respectively. The PCR fragments were purified, digested and ligated into the suicide vector pEX18Gm, which has an *oriT* for conjugation. The resulting plasmid pEX18Gm-Δ*eps273-HI* or pEX18Gm-Δ*eps273-O* was transformed sequentially into *E. coli* SY327 and *E. coli* S17-1 using the CaCl_2_ method. Mating between *P. stutzeri* 273 and *E. coli* S17-1 containing pEX18Gm-Δ*eps273-HI* or pEX18Gm-Δ*eps273-O* was performed at 30 °C for 24 h. Colonies that were grown on LB agar supplemented with Cm (25 μg/mL) and Gm (25 μg/mL) represented single-event recombinant strains that contained vector pEX18Gm-Δ*eps273-HI* or pEX18Gm-Δ*eps273-O* incorporated into the chromosome. An individual colony was selected and incubated overnight at 30 °C with shaking in LB broth containing Cm (25 μg/mL) and Gm (25 μg/mL), then diluted 1:1000 into fresh LB broth and plated onto LB medium supplemented with 10% sucrose and incubated for 48 h at 30 °C. A single colony was selected and re-streaked several times before being replicated onto LB medium supplemented with Gm (25 μg/mL) to confirm sensitivity to gentamicin and loss of the pEX18GM vector. Double recombination events in putative mutants were checked by PCR amplification with primers P1/P4 or P5/P8 (Table S4).

### 4.4. EPS Extraction, Purification, and Analysis

The marine bacterium *P. stutzeri* 273 or its derivatives Δ*eps273-HI* or Δ*eps273-O*, was cultured in glass flasks containing the same amount of LB medium, respectively, and incubated at 28 °C under vigorous agitation for 48 h. Corresponding EPS273 components of *P. stutzeri* 273, or its derivatives Δ*eps273-HI* and Δ*eps273-O*, were purified in parallel according to the methods described previously [13]. Briefly, the anti-biofilm active components were purified by ammonium sulfate precipitation, sequential HiTrap™ Q HP column (GE Healthcare, Chicago, IL, USA), ultra-filtration (100-kDa MW cut-off membrane, Millipore, Billerica, MA, USA) and Hiload™ 16/600 Superdex™ 200 column chromatography(version 28-9893-35, GE Healthcare, Chicago, IL, USA). Total sugar contents of the elution fractions were determined by the phenol-sulfuric acid method using glucose as the standard [49].

### 4.5. Anti-Biofilm Assays

The anti-biofilm activity of purified EPS273 from wild-type or deletion mutants against *P. aeruginosa* PAO1 was detected in 24-well polystyrene microplate using our previous method with minor modification [50]. Briefly, overnight culture of *P. aeruginosa* PAO1 was diluted to OD600 of 0.1 with LB medium, and 950 µL of fresh diluted *P. aeruginosa* PAO1 was incubated statically in 24-well polystyrene plate with or without 50 µL of elution fractions of purified corresponding EPS273 components from *P. stutzeri* 273, or its derivatives Δ*eps273-HI* and Δ*eps273-O*, at 37 °C for 24 h. For quantification of biofilm formation, planktonic bacteria were abandoned, and wells of 24-well polystyrene microplate were rinsed gently with sterile distilled water, air-dried for 10 min, and subsequently stained with 1% crystal violet for 10 min. The crystal violet of the stained biofilm was dissolved in 200 μL of ethanol (95%, volume/volume), then detected at 595 nm.

### 4.6. Colony Morphology on Congo Red (CR) Plates

Colonies on CR plate were grown as previously described with minor modifications [51]. Briefly, colonies of *P. stutzeri* 273, or its derivatives Δ*eps273-HI* or Δ*eps273-O* grown on 2216E plates were streaked onto 2216E plates supplemented with Congo red at a final concentration of 8 μg/mL and incubated at 28 °C for three days. The morphology of the colonies on Congo red plates was checked and recorded under an inverted microscope (NIKON TS100, Tokyo, Japan) equipped with a digital camera.

### 4.7. Biofilm Formation of P. stutzeri 273 and Its Derivatives Δeps273-HI or Δeps273-O

To check the biofilm formation of wild-type and corresponding mutant strains, *P. stutzeri* 273, or its derivative Δ*eps273-HI* or Δ*eps273-O*, was cultured in glass tubes containing the same amount of LB medium, respectively, and incubated overnight at 28 °C under vigorous agitation. Thereafter, the overnight culture was diluted to the initial OD600 of 0.02 with fresh LB medium, and tubes containing the same amount of newly-diluted LB medium were incubated at 28 °C statically for 24 h, 36 h, and 48 h, respectively. The biofilm formation of *P. stutzeri* 273, or its derivatives, was checked and recorded.

### 4.8. Nucleotide Sequence Accession Numbers

The complete genome sequence of *P. stutzeri* 273 has been deposited at GenBank under the accession number CP015641.

## Figures and Tables

**Table 1:** Polysaccharide biosynthetic gene cluster in *P. stutzeri* 273.

Accession Number	Protein Designation	Size (Amino Acids)	Putative Function
WP_064482084.1	EPS273-A	534	Diguanylate cyclase
WP_045428334.1	EPS273-B	492	Sensor kinase
WP_064482085.1	EPS273-C	136	Response regulator
WP_064482094.1	EPS273-M	175	Transcription activator
WP_064482090.1	EPS273-H	405	Glycosyltransferase family 1
WP_064482091.1	EPS273-I	372	Glycosyltransferase family 1
WP_064482093.1	EPS273-L	419	Glycosyltransferase family 1
WP_064482087.1	EPS273-E	332	Glycosyltransferase family 2
WP_045428298.1	EPS273-P	309	Glycosyltransferase family 2
WP_064482086.1	EPS273-D	356	Acetyltransferase
WP_064482092.1	EPS273-J	445	Glycosyl hydrolase
WP_064482803.1	EPS273-K	417	O-antigen ligase
WP_064482095.1	EPS273-N	247	O-antigen production
WP_064482096.1	EPS273-O	736	Tyrosine protein kinase
WP_064482098.1	EPS273-R	393	Aminotransferase DegT
WP_064482088.1	EPS273-F	74	Hypothetical protein
WP_064482089.1	EPS273-G	108	Hypothetical protein
WP_064482097.1	EPS273-Q	508	O-antigen unit translocase or flippase (RfbX/Wzx)

## Supplementary Materials

The following are available online at www.mdpi.com/1660-3397/15/7/218/s1.
