# Supplementary material for: Genome Sequence of Pseudomonas stutzeri 273 and Identification of the Exopolysaccharide EPS273 Biosynthesis Locus
Source: Mar Drugs. 2017 Jul 10;15(7):218. doi: 10.3390/md15070218 (PMC5532660; doi:10.3390/md15070218)
Supplement: Supplementary file 1 [file marinedrugs-15-00218-s001.pdf]

marine drugs

Genome Sequence of *Pseudomonas stutzeri* 273 and Identification of the Exopolysaccharide EPS273 Biosynthesis Locus

Shimei Wu [1], Rikuan Zheng [2,3,4], Zhenxia Sha [1,*] and Chaomin Sun [2,4,*]

[1] College of Life Sciences, Qingdao University, Qingdao, 266071, China; shimeiwu2016@126.com
[2] Key Laboratory of Experimental Marine Biology, Institute of Oceanology, Chinese Academy of Sciences, Qingdao, 266071, China; zhengrikuan15@mails.ucas.ac.cn
[3] College of Earth Science, University of Chinese Academy of Sciences, Beijing, 100049, China
[4] Laboratory for Marine Biology and Biotechnology, Qingdao National Laboratory for Marine Science and Technology, Qingdao, 266071, China
[*] Correspondence: shazhenxia@163.com (Z.S.); sunchaomin@qdio.ac.cn (C.S.); Tel.: +86-532-82898857 (C.S.); Fax: +86-532-82898648 (C.S.)

Table S1. General genome features of *P. stutzeri* 273.

Feature	Chromosome
Genome size (bp)	5,030,940 bp
G+C content (%)	60.78%
rRNAs	12
tRNAs	58
Protein coding genes (cds)	4717
Percentage coding	87.89%

Table S2. Number of genes associated with the general COG functional categories.

Code	Value	Description
A	1	RNA processing and modification
B	1	Chromatin structure and dynamics
C	255	Energy production and conversion
D	34	Cell cycle control, cell division, chromosome partitioning
E	312	Amino acid transport and metabolism
F	74	Nucleotide transport and metabolism
G	162	Carbohydrate transport and metabolism
H	139	Coenzyme transport and metabolism
I	142	Lipid transport and metabolism
J	175	Translation, ribosomal structure and biogenesis
K	234	Transcription
L	182	Replication, recombination and repair
M	189	Cell wall/membrane/envelope biogenesis
N	133	Cell motility
O	156	Posttranslational modification, protein turnover, chaperones
P	211	Inorganic ion transport and metabolism
Q	103	Secondary metabolites biosynthesis, transport and catabolism
R	437	General function prediction only
S	311	Function unknown
T	243	Signal transduction mechanisms
U	108	Intracellular trafficking, secretion, and vesicular transport
V	38	Defense mechanisms

Table S3. Number of genes associated with the general KEGG functional categories.

Function class	Function subclass	Value
	Transport and Catabolism	
	Cell Motility	2
Cellular Processes	Cell Growth and Death	52
	Signaling Molecules and Interaction	6
	Signal Transduction	3
	Membrane Transport	132
Environmental	Translation	299
Information Processing	Transcription	121
	Replication and Repair	97
Genetic Information	Folding, Sorting and Degradation	118
Processing	Infectious Diseases	77
	Xenobiotics Biodegradation and Metabolism	1
Human Diseases	Nucleotide Metabolism	31
Metabolism	Metabolism of Terpenoids and Polyketides	98
	Metabolism of Other Amino Acids	44
	Metabolism of Cofactors and Vitamins	24
	Lipid Metabolism	100
	Glycan Biosynthesis and Metabolism	64
	Enzyme Families	44
	Energy Metabolism	74
	Carbohydrate Metabolism	121
	Biosynthesis of Other	252
	Secondary Metabolites	3
	Amino Acid Metabolism	190
Organismal Systems	Digestive System	2

Table S4. Primers used in this study.

Primer name	Sequence (5′-3′)
P1	AGCGAATTCGGAGGCTTTGCAGGTTTC
P2	ACTGGATCCTATGGCAGAGGGGGTGTA
P3	ACTGGATCCGGTGGTCCATCCCTTGTC
P4	ATCCTGCAGCAGCCTGTTCATTCTTGG
P5	GCAGAATTCCGGGGTTTGGTCTGGATC
P6	GACGGATCCTCCTCAACGGCGTCAAAC
P7	GCAGGATCCCCGAGCAGCGCGAAGAGG
P8	GACAAGCTTGACGCCTCGACGGACAGC
